# Differences in the intrinsic immunogenicity and allergenicity of Bet v 1 and related food allergens revealed by site-directed mutagenesis

**DOI:** 10.1111/all.12306

**Published:** 2013-11-14

**Authors:** A Roulias, U Pichler, M Hauser, M Himly, H Hofer, P Lackner, C Ebner, P Briza, B Bohle, M Egger, M Wallner, F Ferreira

**Affiliations:** 1 Department of Molecular Biology, University of SalzburgSalzburg, Austria; 2 Christian Doppler Laboratory for Allergy Diagnosis and Therapy, Department of Molecular Biology, University of SalzburgSalzburg, Austria; 3 Allergieambulatorium ReumannplatzVienna, Austria; 4 Department of Pathophysiology and Allergy Research, Christian Doppler Laboratory or Immunomodulation, Medical University of ViennaVienna, Austria

**Keywords:** apple allergy, birch pollen-associated food allergies, hazelnut allergy, pollen–food syndrome, protein remodeling

## Abstract

**Background:**

Birch pollen allergies are frequently associated with adverse reactions to various fruits, nuts, or vegetables, described as pollen–food syndrome (PFS) and caused by cross-reactive IgE antibodies primarily directed against Bet v 1. Specific immunotherapy (SIT) represents an effective treatment for inhalant allergies; however, successful birch pollen SIT does not correlate well with the amelioration of concomitant food allergies.

**Methods:**

As vaccine candidates, apple Mal d 1 as well as hazelnut Cor a 1 derivatives were designed by *in silico* backbone analyses of the respective allergens. The proteins were produced by site-directed mutagenesis as fold variants of their parental allergens. Because Mal d 1 and Cor a 1 form cysteine-mediated aggregates, nonaggregative cysteine to serine mutants were also generated. The proteins were characterized physicochemically, immunologically, and in *in vivo* models with or without adjuvant.

**Results:**

The structurally modified proteins showed significantly decreased IgE binding capacity. Notably, both *in vivo* models revealed reduced immunogenicity of the hypoallergenic fold variants. When formulated with alum, the monomeric cysteine mutants induced a similar immune response as the aggregated parental allergens, which is in contrast with data published on Bet v 1.

**Conclusion:**

These findings lead to the suggestion that the Bet v 1 structure has unique intrinsic properties, which could account for its high allergenicity. Obviously, these characteristics are not entirely shared with its food homologues from apple and hazelnut. Thus, it is important to tackle pollen-related food allergies from different angles for the generation of effective vaccine candidates to treat birch PFS.

**To cite this article:** Roulias A, Pichler U, Hauser M, Himly M, Hofer H, Lackner P, Ebner C, Briza P, Bohle B, Egger M, Wallner M, Ferreira F. Differences in the intrinsic immunogenicity and allergenicity of Bet v 1 and related food allergens revealed by site-directed mutagenesis. *Allergy* 2014; **69**: 208–215.

Birch pollen allergies are frequently associated with adverse reactions to various fruits (i.e., pomaceous fruits, stone fruits), hazelnut, vegetables, and legumes and are generally described as pollen–food syndrome (PFS) 1. The symptoms of PFS are mediated by cross-reactive IgE antibodies primarily directed against the major birch pollen allergen Bet v 1 and usually manifest themselves either locally and mildly as oral itching and swelling 2 or, in rare occasions, systemically as urticaria or even anaphylaxis 3. In a recent study including 225 birch pollen-allergic patients from Austria, 73% of the patients reported Bet v 1-associated food allergies. 80% of the food-allergic patients showed hypersensitivity reactions to apple and 59.4% reacted with hazelnut; reactions to other Bet v 1-related food allergens were less frequently reported 4. Apple Mal d 1.0108 and hazelnut Cor a 1.0401 share 55% and 67% sequence identity with Bet v 1, respectively 5,6; however, the Bet v 1-fold seems very conserved within the whole allergen family 7. Successful allergen-specific immunotherapy (SIT) against birch pollinosis does not necessarily lead to a reduction of allergic reactions in concomitant food allergies 8–11, possibly due to the fact that antibody epitopes of Bet v 1 and associated food allergens only converge to a certain degree 12. Recently, it has been shown that continuous consumption of apple can reduce OAS symptoms in allergic individuals; however, this clinical improvement failed to create enduring immunologic effects 13. Of note, there are even reports on patients sensitized to the Bet v 1 allergen family who only experience food-associated allergic symptoms 14. Thus, such data highlight the need for specific treatment of birch pollen-related food allergies. For therapy of Bet v 1-mediated birch pollen allergies, a novel derivative termed BM4 has recently been developed 15. The molecule was generated by computer-aided fold analysis of the Bet v 1 backbone followed by site-directed mutagenesis. This rendered BM4 unable to adopt the Bet v 1-like fold, which abolished IgE binding, and moreover, the activation of antigen-presenting cells and T-cell polarization were changed 16. Using this model, we investigated the effect of structural modifications on the Bet v 1-associated food allergens Mal d 1 and Cor a 1, respectively.

## Material and methods

### Patients and sera

Patients with birch pollen allergy and concomitant adverse reactions toward apple and hazelnut were selected based on typical case history, positive skin prick test, and CAP class >3. Experiments with serum samples from allergic donors were approved by the Ethic Committee of the Medical University of Vienna (no. EK028/2006). Informed written consent was obtained from all subjects included in the study.

### Generation of Mal d 1 and Cor a 1 derivatives

Models of Mal d 1 WT and Cor a 1 WT were generated based on crystal structures of Pru av 1 (1E09) and Bet v 1 (1BV1), respectively, using the software *Modeller* (http://salilab.org/modeller/). *In silico* mutagenesis and Z-score calculations were performed as described 17. Mal d 1 and Cor a 1 variants were generated by PCR amplification of mutated fragments of Mal d 1.0108 (AJ417551) and Cor a 1.0401 (AF136945) using internal mismatch primers (Table S1) as described in the article's online Supporting Information. Mal d 1 variants were cloned into a pET28b vector (Merck KGaA, Darmstadt, Germany) using Nco I and Eco R I restriction sites, while Cor a 1 variants were cloned into a pHIS-Parallel2 vector using Nde I and Xho I restriction sites 18.

### Recombinant production of Mal d 1, Cor a 1, and derivatives thereof

rMal d 1.0108 and rCor a 1.0401 termed Mal d 1 WT and Cor a 1 WT thereafter were produced as previously described 19,20. For the production of allergen derivatives, *Escherichia coli* BL21 Star™ (DE3) cells (Invitrogen Corp, Carlsbad, CA, USA) were transformed with the respective plasmids. Proteins were purified from inclusion bodies using a two-step chromatographic procedure as described in the article's online Supporting Information. Residual bacterial endotoxin was removed from recombinant allergens as described 21. Endotoxin content <3 EU/mg was verified by LAL assay (Associates of Cape Cod Inc., East Falmouth, MA, USA).

### Physicochemical characterization of Mal d 1, Cor a 1, and variants thereof

Purity, identity, secondary structure, homogeneity, and aggregation of recombinant proteins were analyzed as previously described 22. A detailed methodology can be found in the article's online Supporting Information.

### IgE binding, mediator release assays and ANS binding

ELISA as well as mediator release experiments were performed as previously described 15. A brief description of the assays is given in the article's online Supporting Information.

### Endolysosomal degradation assay

Endolysosomes were isolated from JAWS II cells by differential centrifugation as previously described 23. 5 μg protein was digested with 7 μg of isolated microsomal proteins in a final volume of 20 μl containing 100 mM citrate buffer pH 4.8 and 2 mM dithiothreitol for 0, 2, 4, 6, 12, and 24 h at 37°C and stopped by heat denaturation. Qualitative analysis was performed by ESI-QTOF mass spectrometer fitted with a capillary reversed-phase HPLC (Waters Corp, Milford, MA, USA).

### Mice and immunizations

Female BALB/c mice at 8–10 weeks of age (Charles River Laboratories, Wilmington, MA, USA) were immunized with either 5 μg antigen adsorbed to Alu-Gel-S (Serva, Heidelberg, Germany) or 10 μg of antigen in PBS pH 7.4 administered subcutaneously as two 50 μl injections in the back of the animals and boosted on days 14, 21, and 42 or 7, 14, and 21. Sera were collected on days 0, 21, and 49 or 0, 12, and 28, respectively. All animal experiments were conducted according to national guidelines approved by the Austrian Ministry of Science (BMWF-66.012/0011-II/10b/2010).

### Serology and ELISPOT assays

IgG1 and IgE responses were determined by ELISA and β-hexosaminidase release assays, as described 24. IL-4, 5, 10, and 13 and IFN-γ were either assessed using mouse T_H_1/T_H_2/T_H_17/T_H_22 13plex FlowCytomix kit (eBioscience, San Diego, CA, USA) or by ELISPOT, according to kit's manual and as previously described 25.

### Statistical analysis

Statistical differences were determined by paired samples *t*-test, Wilcoxon signed-rank test, and Mann–Whitney U-test, depending on source data. A value of *P *<0.05 was considered statistically significant.

## Results

### Generation of the Mal d 1 and Cor a 1 structural variants

**Figure 1:**
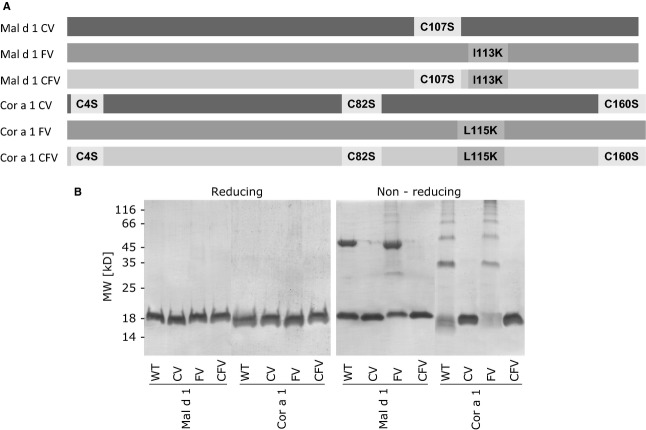
Graphic representation of the mutated residues and their positions on the generated structural variants (A). Coomassie Brilliant Blue-stained reducing and nonreducing SDS-PAGE of Mal d 1, Cor a 1, and mutants thereof (B).

Because no structural data of neither Mal d 1 nor Cor a 1 were available, models were calculated based on protein structures of highly homologous allergens. Using *in silico* mutagenesis, we searched for mutation sites on each of the WT allergens, which would lead to a *Z-*score increase – an indicator for the structural stability of proteins. Thus, mutations giving high *Z-*score values show maximal effect on structural stability. The most destabilizing single-point mutation was selected for each protein, resulting in the generation of Mal d 1 I_113_K and Cor a 1 L_115_K, which were designated Mal d 1 FV and Cor a 1 FV, respectively (Fig.1A).

Similarly to Bet v 1.0102 24, Mal d 1 WT and Cor a 1 WT appeared to form oligomers under nonreducing conditions in SDS-PAGE (Fig.1B). Both allergens contain free cysteine residues; thus, the data suggest disulfide-mediated aggregation. To investigate the effects of oligomerization on the molecules' structural and immunologic behavior, we mutated the cysteins of the WT proteins and their FV mutants to serines. This lead to the production of the cysteine variants (CVs) Mal d 1 C_107_S and Cor a 1 C_4,82,160_S as well as the cysteine fold variants (CFVs) Mal d 1 C_107_S, I_113_K and Cor a 1 C_4,82,160_S, L_115_K (Fig.1A). The recombinant variants were cloned, expressed in *E. coli*, and purified to homogeneity (Fig.1B).

### Structural characteristics of the generated variants

**Figure 2:**
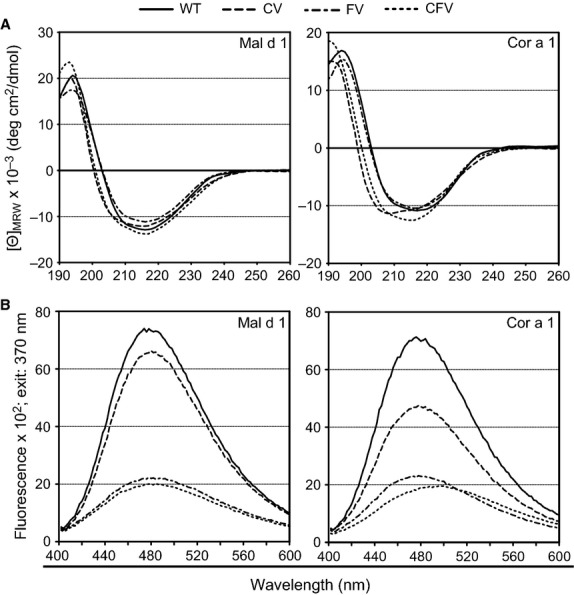
Circular dichroism spectra of the structural variants compared with the respective WT proteins at 20°C. Data are baseline-subtracted, normalized, and presented as mean residue molar ellipticity [Θ]_MRW_ (A). Fluorescence emission spectra of the structural variants compared with the respective WT proteins. Data are baseline-subtracted and presented in fluorescence units (B).

Protein secondary structure was determined by circular dichroism spectroscopy. Far-UV spectra were recorded to compare the structures of allergen variants with their WT counterparts (Fig.2A). CD spectra from all Mal d 1 and Cor a 1 variants were highly similar to their respective WT proteins and resembled the spectra typical for Bet v 1-like proteins. Allergens of the Bet v 1 family, including Mal d 1 and Cor a 1, have highly homologous structures with a characteristic hydrophobic cavity traversing the proteins 26. A wide variety of ligands, one of which is 8-anilino-1-naphthalenesulfonic acid (ANS), were shown to bind to this cavity 27,28. Fluorescence spectroscopy was used to indicate structural alterations in the variants via changes in their ability to bind ANS (Fig.2B). A profound signal decrease was observed for the FVs and CFVs of both Mal d 1 and Cor a 1. The decrease for the CVs was far less significant, albeit stronger for Cor a 1 CV. Altogether, these data indicated an overall preservation of the original protein fold for the WTs and CVs, although a significant structural alteration was observed for FVs and CFVs.

### Cysteine substitutions abrogate the aggregation tendency of Mal d 1 WT, Cor a 1 WT, and the FVs thereof

For detailed aggregation behavior analysis of the variants compared to WT allergens, we performed high-performance size-exclusion chromatography (HPSEC). As shown in Table1, both Mal d 1 and Cor a 1 WTs are prone to form aggregates with monomeric fractions of 56% and 45%, respectively. This aggregation tendency aggravates to an indeterminable level for the FVs. However, for the CVs and CFVs, the Cys to Ser mutations almost lead to an elimination of aggregation tendency, confirmed by dynamic light scattering (DLS) experiments (see Fig. S1 of the article's online Supporting Information).

**Table 1:** Summary of the HPSEC results

Proteins	Monomers	Dimers	Tri-/Tetramers	High MW aggregates	Recovery (%)
Mal d 1	56%	34%	8%	2%	>99
Mal d 1 CV	>98%	<1%	nd	<0.5%	>99
Mal d 1 FV	ws	ws	ws	ws	<15
Mal d 1 CFV	>99%	nd	nd	nd	>99
Cor a 1 WT	45%	28%	18%	9%	>99
Cor a 1 CV	93%	7%	nd	nd	>99
Cor a 1 FV	ws	ws	ws	ws	<16
Cor a 1 CFV	>99%	nd	nd	nd	>99

Recovery, amount of protein eluting from the column relative to the amount expected to be recovered. Low values indicate that high MW aggregates might have bound to column material or parts of HPLC system; nd, not detected; ws, weak signal. Amount of protein reaching the detector was too low to create reading strong enough to allow required calculations.

### FV and CFV mutants display significantly reduced human IgE binding and cross-linking capacity

**Figure 3:**
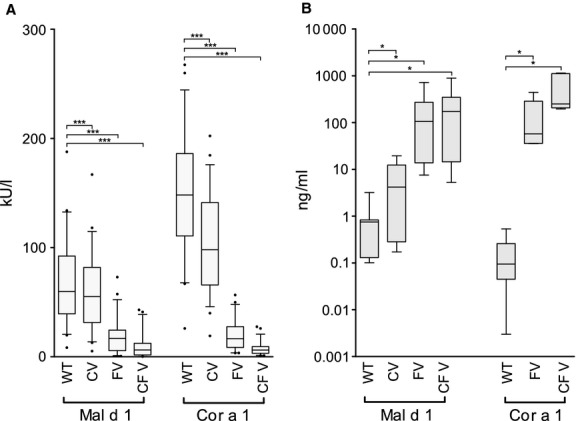
Human IgE ELISA using sera from 50 patients with birch pollen allergy and concomitant OAS. IgE binding of the structural variants was compared with the parental proteins. Data are expressed as IgE concentration (kU/L) ± SEM. *P*-values were calculated with paired samples *t*-test (***P <0.001) (A). RBL assays using patients' sera (*n* = 7). Data are expressed as antigen concentration (ng/ml) needed to trigger 50% mediator release ± SEM. *P*-values were calculated with Wilcoxon signed-rank test (*P <0.05) (B).

The structural variants were tested in ELISA for their ability to bind human IgE antibodies from 50 patients (see Table S2 of the article's online Supporting Information) with birch pollinosis and concomitant oral allergy syndrome (OAS) to apples and/or hazelnuts (Fig.3A). Both FVs and CFVs showed significantly reduced IgE binding capacity compared to the respective WT allergens, the reduction in CFVs being slightly stronger than the one of FVs. Allergenic activity was analyzed by RBL mediator release assays using sera from seven birch pollen-allergic patients (Fig.3B). Although the reaction of each patient to the antigens varied (see Fig. S2 of the article's online Supporting Information), both FVs and CFVs needed >100 times increased antigen concentrations to trigger a 50% mediator release compared with the respective WT molecules. In both assays, also the CVs showed reduced allergenicity, but not as profound as the one of the FVs and CFVs.

### The hypoallergenic variants show reduced immunogenicity in an alum-based *in vivo* model

**Figure 4:**
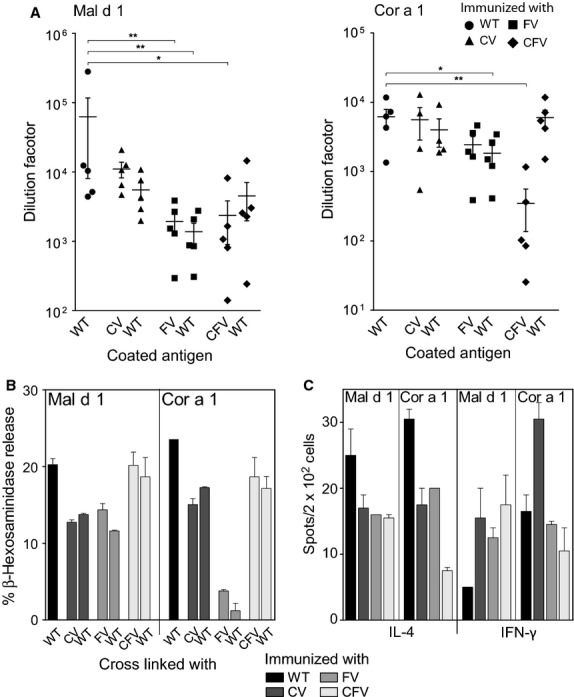
IgG1 antibody responses at day 49 analyzed by ELISA. IgG1 levels of each variant against itself and the WT, compared with the IgG1 response of the respective WT protein. The *y*-axis shows Δ preserum values of the serum dilution at which 50% of the maximum absorption (405 nm) was measured. Each symbol represents sera from one of the five mice immunized with each protein (A). IgE antibody responses by mouse RBL assays. 1 : 40 dilutions of sera pools from mice immunized with each of the antigens were used to passively sensitize mouse RBL cells. Recognition and cross-linking of IgE antibodies was evaluated for the homologous molecules and the WT proteins. Data are expressed as means ± SEM of% mediator release (B). ELISPOT analysis of splenocytes from immunized mice expressed as the mean of cytokine-secreting cells per 2 × 10^5^ cells ± SEM (C). *P*-values were calculated using the Mann–Whitney U-test (*P <0.05; **P <0.01).

To compare the immune responses induced by the WT proteins and their structural variants, we analyzed IgG1 and IgE antibody responses (Fig.4A,B) of BALB/c mice immunized with the respective antigen 1 week after the last booster injection. Both the FVs and CFVs induced significantly lower levels of IgG1 antibodies than the respective WT allergens. Of note, immunization with the FVs and CFVs also resulted in lower induction of antigen-specific IgE antibodies. A reduction in the antibody levels induced by the CVs was also observed, although minimal and not as profound as the one of the FV and CFV mutants.

Using ELISPOT assays, we evaluated the T-cell reactivity for all the mutants and found that immunization with either of them induced IL-4 as well as IFN-γ-secreting T cells (Fig.4C). The IL-4 immune response against all variants was reduced compared to the WT proteins. The levels of IFN-γ-secreting T cells were increased after immunization with the Mal d 1 variants, while in the case of Cor a 1 the IFN-γ response to all variants was comparable to WT. Only Cor a 1 CV induced almost double the amount of IFN-γ-producing T cells. Furthermore, we could observe some difference in the number of IL-5-secreting T cells with an increase for Mal d 1 mutants and the opposite for the Cor a 1 mutants; this trend is not supported by the data obtained with IL-4. Of note, IL-10 levels were all the same for the Mal d 1 proteins, but the two Cor a 1 derivatives FV and CFV completely failed to induce IL-10-producing cells (see Fig. S4 of the article's online Supporting Information). Although no statistical analysis could be performed for RBL as well as ELISPOT assays due to the small number of repetitions, the data showed clear differences between wild-type and mutated allergens.

In an adjuvant-free mouse model, we observed similar immunologic potential with the exception of the FVs, which induced high levels of IgE and T_H_2 cytokines. Detailed analysis of the results is found in the article's online Supporting Information (see Fig. S5).

### Structural variants show the same endolysosomal degradation patterns but different kinetics

Because an antigen's ability to prime T cells and to induce an immune response has recently been linked to its resistance to proteolysis 23, we performed *in vitro* endolysosomal degradation analyses of Mal d 1, Cor a 1, and the structural variants thereof. Analysis of the degradation-derived peptides via tandem mass spectrometry (see Fig. S3 of the article's online Supporting Information) revealed highly similar degradation patterns between the mutants and the respective WT proteins. However, at all time points, degradations of FVs and CFVs resulted in less peptides, while the peptide patterns of CVs reflected the ones of the WT allergens.

## Discussion

Recently, it has been demonstrated that structural alterations of Bet v 1 can not only affect IgE binding but can also improve the immunologic behavior of the protein dramatically 15. Therefore, we investigated whether it is possible to transfer this knowledge to Bet v 1-related food homologues in a generic and unison approach to generate vaccine candidates for the birch pollen-related food allergies. Structural variants of Mal d 1 and Cor a 1, two Bet v 1-food homologues that are commonly associated with birch PFS, were created and extensively characterized.

Three variants of both Mal d 1 and Cor a 1 were generated based on *in silico* mutagenesis of models of each WT allergen using an algorithm, which has been successfully applied to modify Bet v 1.0101. Unlike Bet v 1.0101, both WT Mal d 1 and WT Cor a 1 have free cysteine residues, which form disulfide bridges under physiologic conditions leading to oligomerization of the native allergens. Thus, the cysteins were replaced by serines to mimic the behavior of monomeric Bet v 1. Cysteine and cysteine fold variants were generated, which did not form aggregates any longer. Of note, circular dichroism spectroscopy of the candidate molecules revealed that the serine mutations did not change secondary structure elements. Thus, this approach offers an effective possibility of overcoming disulfide-mediated aggregation of Bet v 1-like allergens.

Next, the immunologic properties of the candidate proteins were investigated. As predicted, the structural alterations of the FVs as well as CFVs significantly decreased their IgE binding capacity as determined by ELISA and mediator release assays. Thereafter, a BALB/c mouse model with alum as adjuvant was established. However, the *in vivo* experiments revealed that the hypoallergenic FVs and CFVs induced a decreased humoral immune response reflected by reduced IgG as well as IgE antibody levels when compared to parental allergens. Recently, an *in vitro* model to predict immunogenicity has been published 23. By digesting proteins with endolysosomal proteases from dendritic cells, conclusions on the immunologic behavior of the antigens *in vivo* can be drawn. Using this method, we found that the fold variants showed a higher susceptibility toward proteolysis, a fact that could explain their low immunogenic potential. This was not expected, given that the Bet v 1 fold variant BM4 was recently shown to display increased resistance toward proteolytic digestion 15. Of note, both CVs appeared stable.

The immune response triggered by the monomeric CVs was comparable to their WT allergens, which formed aggregates. The observation is almost opposite of what has recently been presented by Zaborsky et al. 24 where aggregation of Bet v 1.0102 (formerly Bet v 1.0401) was linked to significantly increased immunogenicity compared to the monomeric Bet v 1 isoform 0101. Moreover, in the absence of alum, the FVs raised a strong T_H_2 response with high IgE titers and significant increase in IL-13 and IL-5 cytokines. In fact, any modifications we made on Mal d 1 and Cor a 1 would always lead to either similar or weaker immune responses showing a drift toward T_H_2. On the contrary, manipulations of Bet v 1a constantly result in stronger responses with a clear T_H_1 bias 15,16,25.

These findings allow us to speculate that Bet v 1 has some unique intrinsic characteristics that are not shared by its food homologues from apple and hazelnut. Thus, manipulation of the Bet v 1-fold does not automatically lead to similar results for any Bet v 1-like allergen. Our results clearly point out that even though the Bet v 1 molecule and birch pollen allergy are intensively studied, it is important to tackle related food allergies from different angles.

## Supporting Information

Data S1 Methods and results.

Figure S1 Aggregation behaviour analysis of Mal d 1, Cor a 1 and their mutants via HPSEC (big graphs) and DLS (small graphs).

Figure S2 Three characteristic RBL titration curves for each of the Mal d 1 (up) and Cor a 1 (down) protein group.

Figure S3 Chronology of peptide cluster formation during *in vitro* endolysosomal degradation.

Figure S4 ELISPOT analysis of splenocytes from immunized mice expressed as the mean of cytokine-secreting cells per 2 × 10^5^ cells ± SEM.

Figure S5 IgG1 antibody responses at day 28 analysed by ELISA.

Table S1 List of primers used for the construction of the Mal d 1 and Cor a 1 structural variants.

Table S2 Patients' sera used within this study.
