# Supplementary material for: Differences in the intrinsic immunogenicity and allergenicity of Bet v 1 and related food allergens revealed by site-directed mutagenesis
Source: Allergy. 2013 Nov 14;69(2):208–15. doi: 10.1111/all.12306 (PMC4041322; doi:10.1111/all.12306)
Supplement: Data S1 — Methods and results. [file all0069-0208-sd1.docx]

**Supporting Information**

**Bet v 1 and its associated food allergens differ in their intrinsic allergy-promoting properties**

Anargyros Roulias^1^, Ulrike Pichler^2^, Michael Hauser^2^, Martin Himly^1^, Heidi Hofer^1^, Peter Lackner^1^, Christof Ebner^3^, Peter Briza^1^, Barbara Bohle^4^ Matthias Egger^2^, Michael Wallner^2^, Fatima Ferreira^2^

^1^Department of Molecular Biology, University of Salzburg, A-5020 Salzburg, Austria

^2^Christian Doppler Laboratory for Allergy Diagnosis and Therapy, Department of Molecular Biology, University of Salzburg, A-5020 Salzburg, Austria

^3^Allergieambulatorium Reumannplatz, Vienna, Austria

^4^Department of Pathophysiology and Allergy Research, Christian Doppler Laboratory or Immunomodulation, Medical University of Vienna, Vienna, Austria

Corresponding Author:

Fatima Ferreira

Christian Doppler Laboratory for Allergy Diagnosis and Therapy, Department of Molecular Biology, University of Salzburg, A-5020 Salzburg, Austria

Hellbrunnerstrasse 34, A-5020 Salzburg, Austria

Tel: +43 (0) 662 8044 5734

Email: Fatima.Ferreira@sbg.ac.at

**Supporting Information**

**METHODS**

**Generation of DNA constructs**

Primers were obtained from Eurofins MWG Operon (Ebersberg, Germany). Plasmid preparations were realized using the Wizard^®^ Plus SV Minipreps DNA purification system (Promega; Madison, WI, US) according to the manufacturer instructions. PCR amplifications were performed with DyNAzyme^TM^ DNA polymerase (Finnzymes; Vantaa, Finland), restriction digests were carried out with restriction endonoucleases from New England Biolabs (Beverly, MA, US) and ligations were completed using T4 DNA ligase from Fermentas (St. Leon-Rot, Germany). Restriction digest and PCR amplification products were always purified by agarose gel electrophoresis using the Wizard^®^ SV Gel and PCR Clean Up system (Promega; Madison, WI, US) following the kit instruction manual.

The structural variants were generated by a two step PCR amplification procedure. In the first step, mutated fragments of the template protein were created by using internal mis-match primer pairs (Table S1) that introduced the desired mutation in the template sequence, and were subsequently gel-purified. In the second step, the mutated DNA fragments were pooled, assembled in a primerless PCR and, finally, the full-length cDNAs were amplified using the according primer pairs (Table S2). The Mal d 1 structural variants were cloned into a pET28b vector from Novagen (Merck KGaA; Darmstadt, Germany) using NcoI and EcoRI restriction sites, while the Cor a 1 variants were cloned into a pHIS-Parallel2 vector ([1](#_ENREF_1)) using the NdeI and XhoI restriction sites. All constructs were sent for sequencing to Eurofins MWG Operon (Ebersberg, Germany).

**Bacterial expression and purification of variants.**

Electrocompetent *E. coli* BL21Star™ (DE3; Invitrogen Corp, Carlsbad, California) were prepared and transformed with the respective constructs. Transformed bacteria were plated on LB agar plates (10 g/L peptone, 5 g/L yeast extract and 5 g/L NaCl and 15 g/L agar) containing 25 mg/L kanamycin (for pET28b constructs) or 100 mg/L ampicillin (for pHIS-Parallel2 constructs) and screened for positive transformants by PCR. For the expression of each of the six mentioned proteins, a single positive transformed colony was picked and inoculated into 2 L of Auto Induction Media (AIM) ([2](#_ENREF_2)) containing 50 mg/L kanamycin (for pET28b constructs) or 200 mg/L ampicillin (for pHIS-Parallel2 constructs). Culture was incubated overnight shaking at 250 rpm at 37 °C. Cells were harvested by centrifugation for 20 min at 5,100 g and 4 °C. Cell pellet was resuspended in 1/15 culture volume of cooled 50 mmol/L TrisBase, 1mmol/L EDTA and 0.1% Triton X-100. Cells were lysed effectively through repeated steps of freezing/thawing, ultrasonication for 20 min and were homogenized with an Ultra-Turrax® disperser. The resulted lysate was centrifuged for 20 min at 20,000 g and 4 °C. The pellet containing cellular debris and insoluble proteins was resuspended in cooled 50 mmol/L TrisBase, 1mmol/L EDTA and 1% Triton X-100, shaken for 20 min at 4 °C and centrifuged again. The previous step of washing the pellet was subsequently repeated using cooled 25% EtOH and 5mmol/L sodium phosphate pH 7.4 buffer. Finally the pellet was resuspended in cooled 6 mol/L urea, 20 mmol/L sodium phosphate pH 7.4 buffer and centrifuged again for 20 min at 20,000 g and 4 °C. The supernatant from this last step was loaded on a 5 ml Q-Sepharose fast flow column (GE Healthcare; Little Chalfont, UK - applies for all the chromatography columns). The protein of interest remained in the flow-through which, after reduction of urea concentration to 4 mol/L and addition of NaCl up to 1.7 mol/L, was loaded on a 5 ml Phenyl-Sepharose column. The protein of interest was eluted from the column applying a 200 ml gradient from the 4 mol/L urea, 1.7 mol/L NaCl and 20 mmol/L sodium phosphate pH 7.4 buffer to a 6 mol/L urea and 20 mmol/L sodium phosphate pH 7.4 buffer collecting 5 ml fractions. Fractions containing pure protein were pooled and gradually dialyzed against a 10 mmol/L sodium phosphate pH 7.4 or a 20 mmol/L TrisBase-HCl pH 9.5 buffer and stored at -20 °C.

**Circular Dichroism**

Analysis of protein secondary structure elements was carried out with a JASCO-J815 spectropolarimeter fitted with a PTC-423S Peltier-type single-position cell holder (Jasco; Essex, UK) using quartz cuvettes of 0.1 cm path length. Far UV (190 to 260 nm) spectra with 0.1 mg/ml protein in appropriate buffers at stable controlled temperature of 20 °C were recorded with 1 nm band width, 1 s response time and 1 nm data pitch. Five consecutive scans were averaged and baseline was subtracted from spectra. Data were presented as mean residue molar ellipticity (Θ_MRW_).

**HPSEC-TDA and DLS**

To evaluate the proteins’ homogeneity, high-performance size-exclusion chromatography was performed using a 7.8 x 300 mm TSKgel G2000SWXL column protected by a 6 x 40 mm guard column (Tosoh Bioscience, Stuttgart, Germany) on a HP1100 analytical chromatography system (Hewlett-Packard, San Jose, CA, US) equipped with a built-in UV detector and online coupled with a right-angle light scattering, refractive index and viscosity detector array (TDA302; Viscotek, Houston, TX, USA). Size exclusion chromatography triple detection runs were performed at 0.5 ml/min in appropriate buffers. The molecular weight and hydrodynamic radius of eluting peaks were determined using a combination of data obtained by sequential UV (280 nm), refractive index, intrinsic viscosity, and right-angle light scattering detection. Detector calibration was performed using bovine serum albumin from Sigma (A7638) weighed out at 1.0 mg/ml.

Aggregation behaviour of proteins in solution was assessed by means of dynamic light scattering using a DLS 802 system (Viscotek Corp.; Houston, TX, US) at concentrations ranging from 0.39-1.89 mg/ml and appropriate buffer conditions, after 10 min centrifugation at 14000 g. The solvent settings for water were used. Data were accumulated for 10 x 10 sec and the correlation function was fitted into the combined data curve, from which the intensity distribution was calculated ([3](#_ENREF_3)). The determined intensity distribution was weighted statistically by mass using the mass model for proteins (OmniSizeTM) displaying the hydrodynamic radius and polydispersity.

**ELISA experiments**

To determine the human IgE binding capacity of the proteins enzyme linked immunoabsorbent assays (ELISA) were performed. Proteins (100 ng/well in 50 μl PBS) were coated on 96-well Maxisorp plates (Nalge Nunc International; Rochester, NY, US) overnight at 4 °C. Plates were blocked with TBS pH 7.4, 0.05% (vol/vol) Tween, 1% (vol/vol) BSA for 2 h at RT, and incubated with human sera diuted 1:10 overnight at 4 °C. Detection was based on alkaline phophatase-conjugated monoclonal anti-human IgE antibodies (Beton Dickinson Bioscienses, NJ, US) after incubation for 90 min at 37 °C followed by 90 min at 4 °C. Measurements were carried out with a TECAN Sunrise™ microplate reader (Tecan group Ltd; Männerdorf, Switzerland) at a wavelength of 405/492 nm using 10 mmol/L of 4-Nitrophenyl phosphate (Sigma-Aldrich®; St. Louis, MO, US) as substrate. An internal standard was used in each plate for means of data normalising. Measurements were performed in duplicates and the mean of the OD values was transformed to IgE concentration, based on a standard curve generated from analysis of patients with known amounts of specific IgE antibodies.

**β-Hexosaminidase release assays**

In order to assess the allergenic potential of the proteins, a rat basophile leukaemia (RBL) cell mediator release assay was carried out, using a cell line (RBL-2H3) transfected with the human FcεRI receptor ([4](#_ENREF_4)) enabling the binding of human IgE from allergic patients’ sera. 10^5^ cells/well were aliquoted in 96-well tissue culture plates (Nalge Nunc International; Rochester, NY, US) and sensitized with patients’ complement-inactivated sera of a final dilution 1:5 overnight at 37 °C, 7% CO_2_. Cells were washed (Tyrode’s buffer, 0.1% BSA) and were subsequently incubated with nine (9x) serial 1:10 antigen dilutions starting with 100 μg/ml in Tyrode’s buffer, 50% D_2_O for 1h at 37 °C, 7% CO_2_ to induce cross-linking. Antigen specific β-hexosaminidase release in the supernatant was measured upon enzymatic cleavage of 4-Methylumbelliferyl N-acetyl-β-D-glucosaminide (Sigma-Aldrich®; St. Louis, MO, US) in 100 mmol/L citrate buffer pH 4.5. Total cellular β-Hexosaminidase release values were calculated from cells treated with Triton X-100 (Sigma-Aldrich®; St. Louis, MO, US). Fluorescence measurement was performed with a TECAN GENios™ multifunction fluorescence, absorbance and luminescence microplate reader at an excitation wavelength of 360 nm and an emission wavelength of 465 nm. Data were evaluated with the Tecan XFluor^TM^ software and values were expressed as percentage of the Triton treated cells release.

**ANS binding**

Binding analyses of 8-anilino-1-naphtalenesulphonic acid (ANS) on recombinant proteins were performed using 10 μmol/L of protein and 50 μmol/L of ANS in a 10 mM NaP pH 7.4 buffer. Measurements were performed on a TECAN Infinite^®^ 200 PRO multimode microplate reader (Tecan group Ltd; Männerdorf, Switzerland), applying an excitation wavelength of 370 nm and scanning emission from 410 nm to 600 nm with 2 nm steps ([5](#_ENREF_5)).

**RESULTS**

**Assessing the mutants' immunogenicty in the absence of adjuvants**

In order to investigate the immunologic behaviour of our candidate proteins avoiding effects of collateral aggregation induced by protein absorbance to Alum, we established an adjuvant-free mouse model. The pattern of the humoral immune response (IgG1 and IgE) was similar as observed with the Alum-model. In general, for all variants antibody titers were decreased when compared to the WT allergens. Exceptions were the similar IgG1 response of Cor a 1 FV and the significantly increased IgE levels of both Mal d 1 and Cor a 1 FVs compared to the WT proteins. In terms of T-cell reactivity, both CVs failed to induce substantial cytokine production, whereas the FVs and CFVs, respectively, showed a significant increase in IL-13 and IL-5 levels. Of note, IFN-γ was almost doubled for the FVs and slightly reduced for the CFVs, compared to WT allergens.

**FIGURE AND TABLE LEGENDS**

Figure S1. Aggregation behaviour analysis of Mal d 1, Cor a 1 and their mutants via HPSEC (big graphs) and DLS (small graphs). No results are shown for the FVs since both molecules aggregated heavily rendering it impossible to obtain quality data. HPSEC: High performance size exclusion chromatography; DLS: Dynamic light scattering; RI: Refractive index; R_H_: Hydrodynamic radius.

Figure S2. Three characteristic RBL titration curves for each of the Mal d 1 (up) and Cor a 1 (down) protein group.

Figure S3. Chronology of peptide cluster formation during *in vitro* endolysosomal degradation. Peptide clusters obtained after 2, 4, 6, 12, and 24 hours of proteolytical digestion were sequenced by means of mass spectrometry. Each coloured horizontal bar represents a single peptide generated by proteolysis. The position (amino acid number) of each peptide in the protein sequence is indicated by the numbers on the upper part of the image.

Figure S4. ELISPOT analysis of splenocytes from immunized mice expressed as the mean of cytokine-secreting cells per 2 x 10^5^ cells ± SEM.

Figure S5. IgG1 antibody responses at day 28 analysed by ELISA. IgG1 levels of each variant against itself and the WT, compared with the IgG1 response of the respective WT protein. The y-axis shows Δ pre-serum values of serum antibody titers. Each symbol represents sera from one of the five mice immunized with each protein (A). IgE antibody responses by RBL assays. 1:20 dilutions of sera pools from mice immunized with each of the antigens were used to passively sensitize RBL cells. Recognition and cross linking of IgE antibodies was evaluated for the homologous molecules and the WT proteins. Data are expressed as means ± SEM of % mediator release (B). Secreted cytokine levels analysis of splenocytes from immunized mice expressed as means of pg/ml ± SEM (C). *P* values were calculated with the Mann-Whitney U test (**P* < .05; ***P* <.01).

Table S1. List of primers used for the construction of the Mal d 1 and Cor a 1 structural variants. Exchanged bases are shown in bold; restriction sites are underlined.

Table S2. Patient sera used within this study. **AS**: Asthma, **Po**: pollinosis. **Ap**: apple, **Nu**: hazelnut, **Ro**: Rosaceae fruits, **Ki**: kiwi, **Pe**: pear, **Se**: celery, **O**: other and **ns**: unspecified PFS.

**REFERENCES**

1. Sheffield P, Garrard S, Derewenda Z. Overcoming Expression and Purification Problems of RhoGDI Using a Family of “Parallel” Expression Vectors. *Protein Expression and Purification* 1999 Feb;**15**(1):34-39.

2. Studier F. Protein production by auto-induction in high density shaking cultures. *Protein Expression and Purification* 2005 May;**41**(1):207-234.

3. Himly M, Nony E, Chabre H, Van Overtvelt L, Neubauer A, Van Ree R, et al. Standardization of allergen products: 1. Detailed characterization of GMP-produced recombinant Bet v 1.0101 as biological reference preparation. *Allergy* 2009 Jul;**64**(7):1038-1045.

4. Vogel L, Lüttkopf D, Hatahet L, Haustein D, Vieths S. Development of a functional in vitro assay as a novel tool for the standardization of allergen extracts in the human system. *Allergy* 2005 Aug;**60**(8):1021-1028.

5. Vaz DC, Rodrigues JR, Sebald W, Dobson CM, Brito RM. Enthalpic and entropic contributions mediate the role of disulfide bonds on the conformational stability of interleukin-4. *Protein Sci* 2006;**15**(1):33-44.
