# Supplementary figures and images for: Differences in the intrinsic immunogenicity and allergenicity of Bet v 1 and related food allergens revealed by site-directed mutagenesis
Source: Allergy. 2013 Nov 14;69(2):208–15. doi: 10.1111/all.12306 (PMC4041322; doi:10.1111/all.12306)

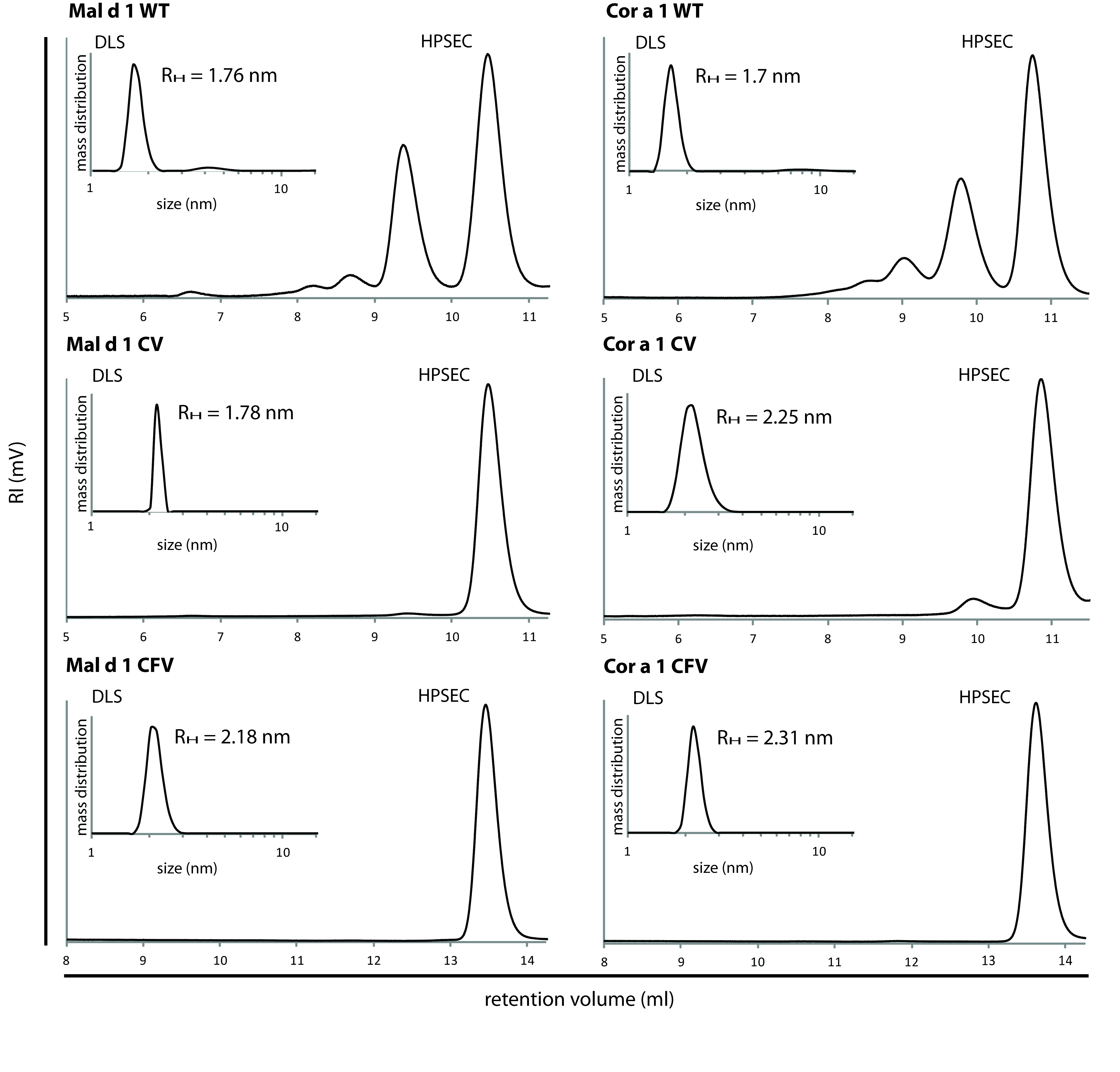

Supplement: Figure S1 — Aggregation behaviour analysis of Mal d 1, Cor a 1 and their mutants via HPSEC (big graphs) and DLS (small graphs). [file all0069-0208-sd2.tif]

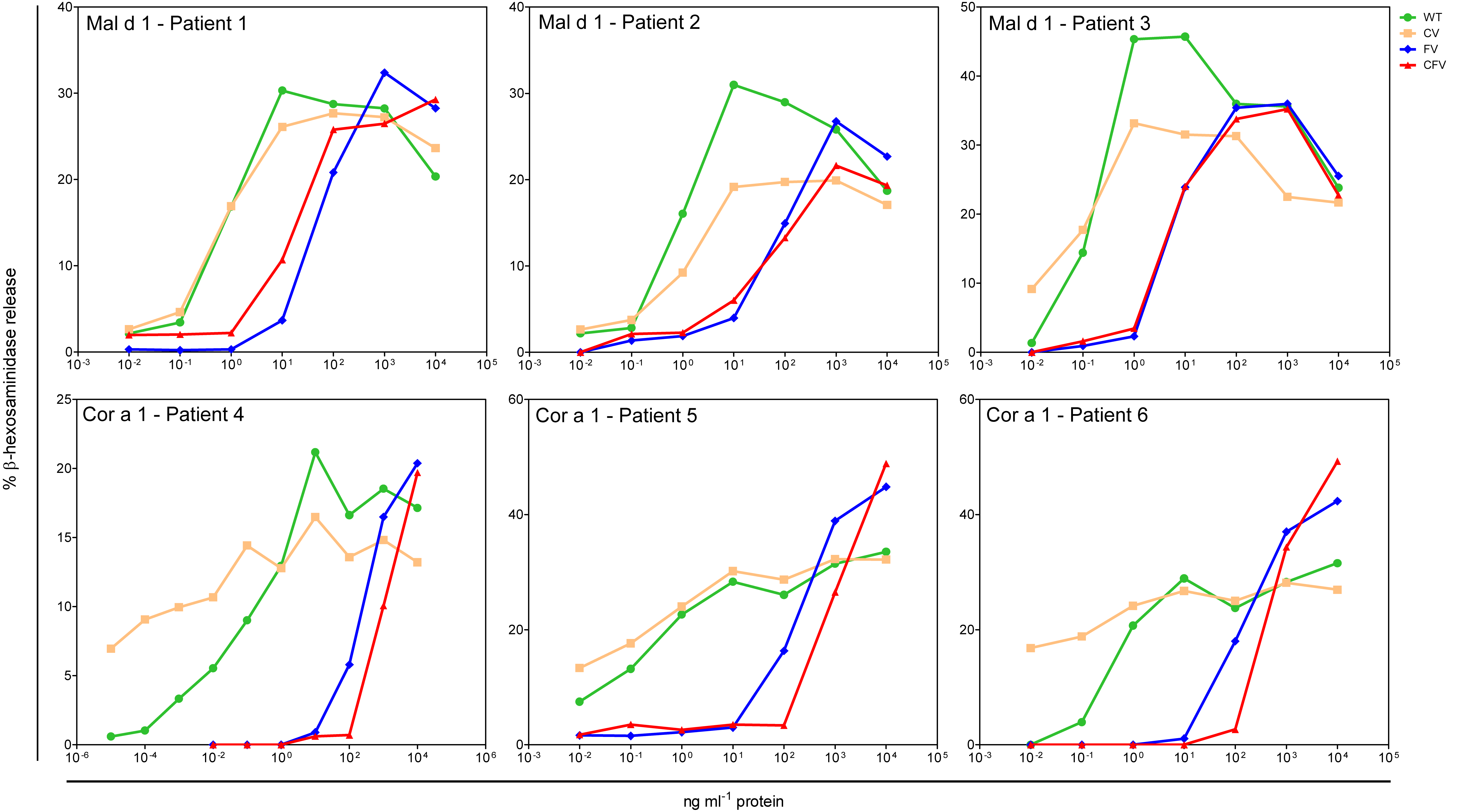

Supplement: Figure S2 — Three characteristic RBL titration curves for each of the Mal d 1 (up) and Cor a 1 (down) protein group. [file all0069-0208-sd3.tif]

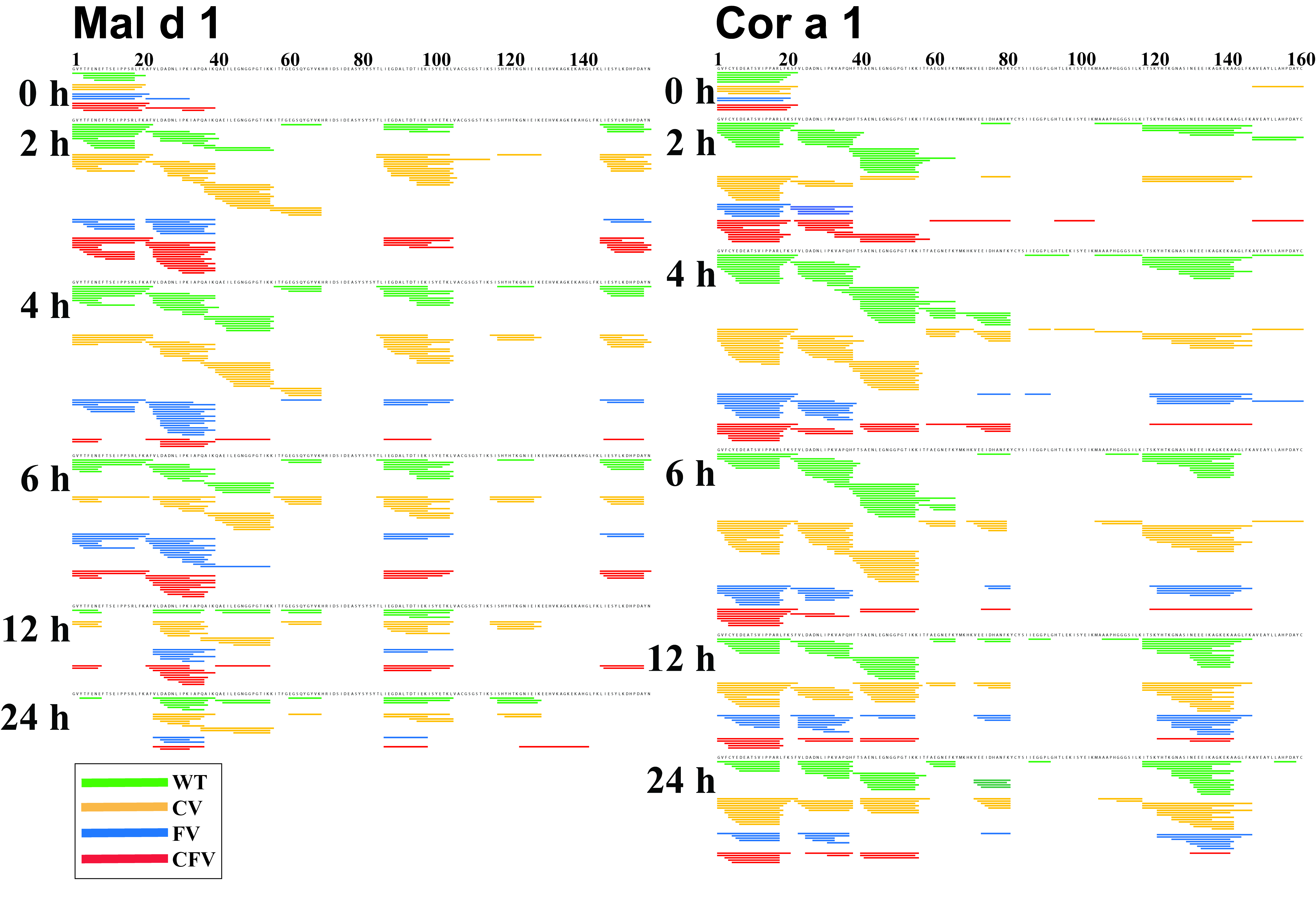

Supplement: Figure S3 — Chronology of peptide cluster formation during in vitro endolysosomal degradation. [file all0069-0208-sd4.tif]

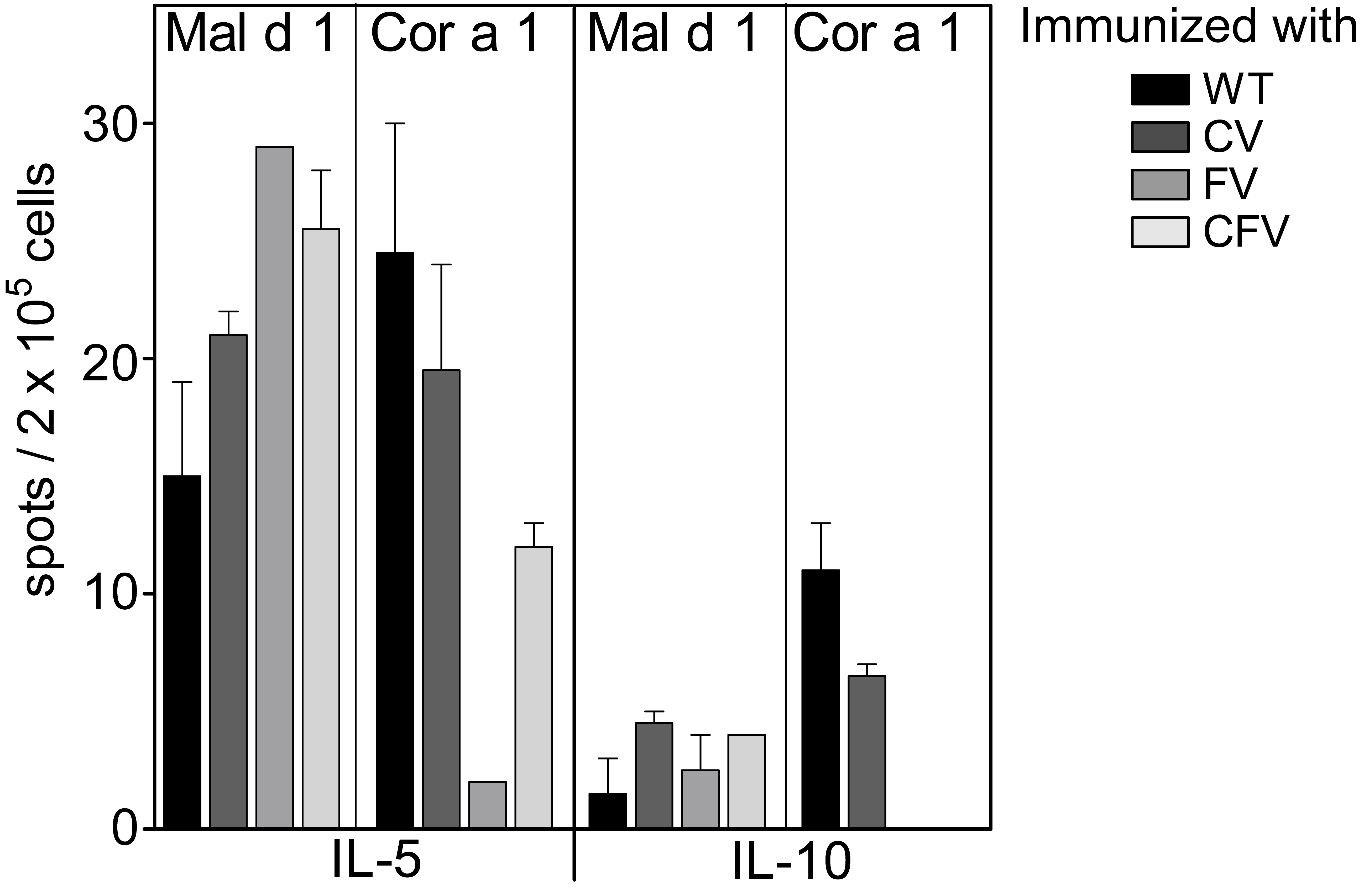

Supplement: Figure S4 — ELISPOT analysis of splenocytes from immunized mice expressed as the mean of cytokine-secreting cells per 2 × 10^5^ cells ± SEM. [file all0069-0208-sd5.tif]
