# Supplementary material for: Differences in the intrinsic immunogenicity and allergenicity of Bet v 1 and related food allergens revealed by site-directed mutagenesis
Source: Allergy. 2013 Nov 14;69(2):208–15. doi: 10.1111/all.12306 (PMC4041322; doi:10.1111/all.12306)
Supplement: Table S1 — List of primers used for the construction of the Mal d 1 and Cor a 1 structural variants. [file all0069-0208-sd7.docx]

Table S1. List of primers used for the construction of the Mal d 1 and Cor a 1 structural variants. Exchanged bases are shown in bold; restriction sites are underlined.

| **Ref.** | **Primer** | **Sequence** |
| --- | --- | --- |
| A | Mal Fwd Nco I | 5'-agggcgccatgggtgtgtacacct-3' |
| B | Mal Rev EcoR I | 5'-cctttgaattcttaggtgtaggcat-3' |
| C | Mal FV Fwd | 5'-agtggttccacc**aaa**aagagcatc-3' |
| D | Mal FV Rev | 5'-gatgctctt**ttt**ggtggaaccact-3' |
| E | Mal CV Fwd | 5'-agttggtggca**agc**ggaagtggttc-3' |
| F | Mal CV Rev | 5'-gaaccacttcc**gct**tgccaccaact-3' |
| G | Cor Fwd Nde I | 5'-ggcccatatgggtgttttctgcta-3' |
| H | Cor Rev Xho I | 5'-tcggctcgagttaacagtaggcatcagggtgtg-3' |
| I | Cor FV Fwd | 5'-gaggatccatc**aaa**aagatcaccag-3' |
| J | Cor FV Rev | 5'-ctggtgatctt**ttt**gatggatcctc-3' |
| K | Cor CV Fwd Nde I | 5'-ggcccatatgggtgttttc**agc**tacga-3' |
| L | Cor CV Rev Xho I | 5'-tcggctcgagtta**gct**gtaggcatcagggt-3' |
| M | Cor CV Fwd | 5'-acttcaaatac**agc**tacagcatcat-3' |
| N | Cor CV Rev | 5'-atgatgctgta**gct**gtatttgaagt-3' |
