# Supplementary material for: Differences in the intrinsic immunogenicity and allergenicity of Bet v 1 and related food allergens revealed by site-directed mutagenesis
Source: Allergy. 2013 Nov 14;69(2):208–15. doi: 10.1111/all.12306 (PMC4041322; doi:10.1111/all.12306)
Supplement: Table S2 — Patients' sera used within this study. [file all0069-0208-sd8.docx]

Table S2. Patient sera used within this study. AS: Asthma, Po: pollinosis. Ap: apple, Nu: hazelnut, Ki: kiwi, Pe: pear, Se: celery, O: other and ns: unspecified PFS.
